# Intermittent Auscultation for Intrapartum Fetal Heart Rate Surveillance

**DOI:** 10.1111/jmwh.70079

**Published:** 2026-02-26

**Authors:** Kristen Ostrem‐Niemcewicz, Jessica Holm, Lisa Kane Low

**Affiliations:** ^1^ American College of Nurse‐Midwives Washington, DC; ^2^ Oregon Health and Science University Portland Oregon; ^3^ Hennepin Healthcare Minneapolis Minnesota; ^4^ School of Nursing, University of Michigan Ann Arbor Michigan

**Keywords:** electronic fetal monitoring, fetal heart rate, intermittent auscultation, intrapartum fetal surveillance

## Abstract

Fetal heart rate surveillance is a standard component of intrapartum care. The fetal heart rate can be evaluated using intermittent auscultation or electronic fetal monitoring. Research that has compared these 2 strategies found them to be equivalent with respect to long‐term neonatal outcomes. The purpose of this clinical bulletin by the American College of Nurse‐Midwives is to review evidence for the use of intermittent auscultation and provide recommendations for intermittent auscultation technique, interpretation, and documentation.

## INTRODUCTION

Standard evaluation of fetal well‐being during labor includes periodic assessment of the fetal heart rate (FHR), FHR pattern, and FHR response to intrapartum stimuli and events. Effective methods of evaluation and meaningful interpretation of FHR data have long been subjects of clinical debate.^1^, ^2^, ^3^ Continuous electronic fetal monitoring (EFM) is the most common method of intrapartum fetal surveillance in current use, but this method has not been shown to be more effective than intermittent auscultation (IA).^4^ The use of EFM has also been associated with an increased risk for cesarean birth and operative vaginal delivery without improved outcomes in the setting of low‐risk pregnancies.^4^, ^5^, ^6^ The type of fetal well‐being assessment during labor should be chosen based on the risk status and preferences of the birthing person, and it has been recommended that the chosen method should be the least‐invasive one possible to promote physiologic labor and birth.^7^

The recommendations of most professional organizations regarding FHR assessment during labor are based upon protocols used in randomized controlled trials (RCTs) in which the authors compared IA and EFM.^7^, ^8^, ^9^, ^10^ Guidelines based on evidence‐based application of IA during labor are limited in availability. This clinical bulletin reviews the evidence for the use of IA and presents recommendations for use that are based on the best available scientific data.

## INTERMITTENT AUSCULTATION

IA is a method of FHR surveillance that involves counting the FHR for a specified amount of time at specified intervals in relation to uterine contractions. IA may be done with a fetoscope, which uses bone conduction to assist in hearing the opening and closing of the valves of the fetal heart, or with a handheld Doppler ultrasound, which is used to detect fetal heart motion and convert it to sound.

Researchers who have evaluated the reliability and validity of IA have not reported consistent results.^11^, ^12^, ^13^ However, it appears that IA using a multiple‐count strategy to assess the FHR during and after a contraction can be used to reliably detect heart rate, rhythm, accelerations, and decelerations,^11^, ^14^, ^15^ but it cannot be used to differentiate between types of decelerations or to determine baseline variability with accuracy (Table 1).^11^, ^12^, ^14^, ^15^, ^16^, ^17^, ^18^, ^19^, ^20^ Despite these limitations, researchers who have compared the 2 fetal surveillance strategies found them to be equivalent with respect to long‐term neonatal outcomes.^4^, ^21^, ^22^, ^23^ Therefore, the inability to consistently determine the FHR variability or type of deceleration in labor using IA does not appear to be clinically significant when monitoring patients in labor who are at low risk for uteroplacental insufficiency or who have fetuses with normal baseline heart rates.^24^ To date, no trials have been published evaluating IA protocols. In a systematic review by Blix and others (2019), no evidence was found to recommend a Doppler device instead of the Pinard device for IA, or vice versa.^3^

**Table 1:** Fetal Heart Rate Characteristics Determined via Auscultation Versus Electronic Monitor

FHR Characteristic^a^	Fetoscope	Doppler Without Paper Printout	Electronic FHR Monitor
Variability	No	No	Yes
Baseline rate	Yes	Yes	Yes
Accelerations	Detects increases^b^	Detects increases^b^	Yes
Decelerations	Detects decreases	Detects decreases	Yes, and differentiates between types of decelerations
Rhythm^c^	Yes	Yes	Yes
Double counting or half‐counting FHR	No, excepting user error	May double count or half count	May double count or half count
Differentiation of maternal and fetal heart rate	Yes	May detect maternal heart rate	May detect and record maternal heart rate

Abbreviation: FHR, fetal heart rate.

a Definitions of each FHR characteristic are based on those reported by Macones et al.^25^

b Per the method described by Paine et al (Part I)^17^ and Paine et al (Part II).^18^

c Defined as regular or irregular. None of these devices can diagnose the type of fetal arrhythmia.

Source: Adapted from Drummond S, Rust C. Techniques for fetal heart and uterine activity assessment. In: Lyndon A, Ali LU, eds. *Fetal Heart Monitoring Principles and Practices*. 6th ed. Kendall‐Hunt Publishing; 2021:84‐90.^16^ Used with permission from the Association of Women's Health, Obstetric and Neonatal Nurses.

## ELECTRONIC FETAL MONITORING

With EFM, an ultrasound or an electrode records the fetal electrocardiogram to produce an auditory and visual representation of the FHR that is continuously recorded as the FHR tracing. Continuous EFM is used to detect and document the baseline rate, FHR variability, accelerations, and periodic or episodic decelerations. The presence of moderate FHR variability, accelerations, and a normal baseline rate is highly predictive of a well‐oxygenated fetus at the time of observation.^25^, ^26^ However, the relationship between variant FHR patterns and fetal acid‐base status is highly variable.^27^ Most variant FHR patterns have a low positive predictive value for identifying fetal acidemia.^28^

## RESEARCH COMPARING INTERMITTENT AUSCULTATION AND ELECTRONIC FETAL MONITORING DURING LABOR

Since the adoption of continuous EFM as the standard of care, multiple RCTs and subsequent meta‐analyses designed to determine the effectiveness of EFM have been performed.^4^, ^21^, ^22^, ^23^ The *Cochrane Database of Systematic Reviews* published a meta‐analysis of 13 RCTs in which investigators compared maternal and neonatal outcomes after continuous EFM versus IA during labor for more than 37,000 birthing people.^4^ The people in the continuous EFM group had an increase in cesarean births (relative risk [RR], 1.63; 95% CI, 1.29‐2.07; n  =  18,861; 11 trials) and operative vaginal births (RR, 1.15; 95% CI, 1.01‐1.33; n = 18,615; 10 trials) but no difference in perinatal mortality (RR, 0.86; 95% CI, 0.59‐1.23; n = 33,513; 11 trials) or rates of cerebral palsy (RR, 1.75; 95% CI, 0.84‐3.63; n = 13,252; 2 trials).^4^ There was a 50% decrease in the incidence of neonatal seizures in the continuous EFM group compared with the IA group (RR, 0.50; 95% CI, 0.31‐0.80; n = 32,386; 9 trials).^4^ However, in a follow‐up study of the infants who had seizures at 4 years of age, the researchers found an equal number of children in each group with cerebral palsy, leading them to conclude that continuous EFM offers little, if any, benefit.^27^, ^29^ In practice, the reduction in the incidence of neonatal seizures must be balanced against the morbidity associated with cesarean and operative vaginal births. Cesarean birth significantly increases the incidence of maternal morbidity and mortality compared with that with vaginal birth.

Use of EFM as an initial admission test for laboring women has also been studied in 5 RCTs and a subsequent meta‐analysis of more than 13,000 low‐risk people in labor.^30^, ^31^, ^32^, ^33^, ^34^ The EFM admission test is usually a 20‐minute continuous recording of the FHR that is evaluated for any characteristics that may increase the risk for fetal acidemia. Use of an EFM admission test increased the incidence of continuous EFM during labor (RR, 1.30; 95% CI, 1.14‐1.48; n  =  10,753; 3 trials), and there was a trend toward an increase in the rate of cesarean birth (RR, 1.20; 95% CI, 1.00‐1.44; n = 11,338; 4 trials).^30^ There were no significant differences in other labor interventions such as amniotomy, labor augmentation, or epidural analgesia. Similarly, there were no differences between the groups in adverse neonatal outcomes. The authors concluded that continuous EFM as a labor admission test has no clear benefit compared with IA in birthing persons with no known risks for fetal acidemia at the onset of spontaneous labor at term.^30^

## METHOD OF INTERMITTENT AUSCULTATION

IA can be used to determine the baseline FHR and detect periodic FHR changes. Most professional associations have made recommendations on the frequency of IA based on protocols used in RCTs comparing EFM and IA.^7^, ^8^, ^9^, ^10^ However, the timing of IA with regard to uterine contractions must also be considered. Romano and Buxton (2020) demonstrated the importance of shared education and IA practice using simulation to promote consistency in the method and clinical application of IA.^35^

### Evaluation of Baseline Fetal Heart Rate

To determine the baseline FHR, the FHR is auscultated between contractions and when the fetus is not moving. At the same time, the birthing person's radial pulse is palpated to ensure that the FHR, not the birthing person's heart rate, is auscultated.^9^, ^16^ After establishing the baseline rate, the FHR is auscultated for 15 to 60 seconds at recommended intervals between contractions and when the fetus is not moving.

### Evaluation of Periodic FHR Changes

Listening for increases and decreases in FHR is the second component of IA. FHR increases and decreases are generally thought to correlate with accelerations and decelerations noted on EFM. Typically, the health care provider auscultates the FHR for a period of time (eg, 15–60 seconds) and notes any audible increase or decrease in rate. Methods have been devised to validate this information^13^, ^14^, ^15^, ^17^, ^18^ based on studies primarily done with pregnant people who were not in labor or via the use of simulated data such as FHR recordings.^19^ Therefore, although the techniques may be used in the intrapartum setting, they have not been validated for use in the intrapartum setting. In most of the studies, investigators used a multiple‐count strategy whereby the observer counted the FHR during several 5‐ to 15‐second increments.^15^, ^17^, ^18^, ^19^, ^20^ An increase in the number obtained from each 5‐ or 15‐second count in subsequent intervals indicated an acceleration, and a decrease in the rate indicated a deceleration. These rates can be plotted on a graph for documentation, as described in 2 studies by Paine et al.^17^, ^18^ If there is a question as to whether accelerations or decelerations have been auscultated, continued auscultation throughout a uterine contraction may provide clarification. The multiple‐count strategy is likely to be more accurate than a single‐count strategy.^19^

### Frequency of Intermittent Auscultation

To date, investigators have not determined the optimal frequency of IA during labor. The current recommendations of professional organizations representing perinatal health care providers are summarized in Table 2. In the absence of evidence‐based parameters to define the optimal interval for auscultation, an interval ranging from every 15 to 30 minutes during the active phase; every 15 minutes during the second stage, prior to expulsive efforts; and every 5 minutes after initiation of pushing may be reasonable as long as the auscultated FHR is normal and there are no other labor characteristics that would suggest a need for more‐frequent monitoring.

**Table 2:** Recommendations of Professional Organizations for Frequency of Fetal Heart Rate Auscultation for Laboring People Who Are Low Risk^a^ During Labor

Organization	Latent Phase	Active Phase, min	Second Stage, min
American College of Nurse‐Midwives		15–30	5‐15
Association of Women's Health, Obstetric and Neonatal Nurses	Insufficient evidence; at discretion of health care provider	15‐30	5‐15^b^
American College of Obstetricians and Gynecologists and American Academy of Pediatrics Joint Guidelines for Perinatal Care	Insufficient evidence; use best clinical judgment	15	5
Royal College of Obstetricians and Gynaecologists		15^d^	5^c^
Society of Obstetrics and Gynaecologists of Canada^d^	At least hourly once admitted to hospital unit	15‐30	At least every 5 or immediately following each contraction^e^

Abbreviation: IA, intermittent auscultation.

a No guidelines for the above‐listed professional organizations specifically define low risk. This bulletin defines low risk as birthing people who have no medical or obstetric conditions that are associated with uteroplacental insufficiency or conditions that are associated with an increased incidence of umbilical artery pH of less than 7.1 at birth.

b Every 15 minutes with complete dilation and passive fetal descent, and every 5 to 15 minutes during active pushing.

c For a minimum of 60 seconds after a contraction.

d IA should be performed by individuals who have received initial and ongoing education and are experienced in the technique of IA, which includes palpation of contractions and recognition of fetal heart rate patterns via auscultation.

e When pushing has been initiated.

The frequency of auscultation should be individualized based upon the contraction pattern, level of maternal activity, and interventions that may affect the FHR. In addition to listening to the FHR with IA at regular intervals, it is recommended that the FHR be assessed before and after vaginal examinations or rupture of membranes.^16^

### Timing of Auscultation in Relation to Uterine Contractions

The timing of auscultation varies among protocols but can include auscultation throughout and after contractions or between contractions.^7^, ^36^, ^37^ Evidence for the best technique is limited. Although one can listen and count the FHR throughout a contraction and for a short time after the contraction resolves, this is technically difficult to do. One of the primary goals of listening throughout the contraction and for a brief time after the contraction ends is to ensure that the listener can detect periodic FHR decelerations if they are present. If one listens between contractions only, there is the potential to miss clinically important decelerations that can increase the risk for fetal acidemia. Thus, the recommended technique for detecting decelerations is to listen during the last portion of a uterine contraction and for a short period after the contraction is complete. For this purpose, a Doppler with speaker has advantages over a fetoscope, including ease of use in several positions of the birthing person and during water immersion. Audibility allows for validation and collaboration with the birthing person and other members of the caregiving team. The recommended technique for IA is provided in Table 3.

**Table 3:** Techniques for Performing Intermittent Auscultation

1. Perform Leopold's maneuvers to identify the fetal presentation and position.
2. Assist the birthing person into a position that maximizes audibility and preserves comfort.
3. Assess the frequency and duration of uterine contractions.
4. Determine the pulse rate of the birthing person.
5. Place the fetoscope or Doppler over the fetal thorax or back.
6. Determine the baseline FHR by listening between contractions and when the fetus is not moving. Verify the birthing person's pulse rate.
7. Subsequently, count the FHR starting at the peak of the uterine contraction and for a short period of time after the contraction resolves every 15–30 min in active labor and every 5–15 min in the second stage of labor (see Table 2).
8. Note increases (accelerations) or decreases (decelerations) from the baseline rate by counting and recording the FHR using a multiple‐count strategy agreed upon by practice protocol.^a^

Abbreviation: FHR, fetal heart rate.

a Several multiple‐count strategies have been tested in studies of auscultation. Note that in some of these studies, researchers evaluated FHR auscultation for nonstress tests, and the particular technique has not been tested for intrapartum use. Details of previous testing can be found in Miller et al,^15^ Paine et al (Part I),^17^ Paine et al (Part II),^18^ Schifrin et al,^19^ and Daniels and Boehm.^20^

## INTERPRETATION OF THE FETAL HEART RATE DURING LABOR

The purpose of monitoring the FHR during labor is to detect the development of clinically significant metabolic acidemia in the fetus. If the fetus does not have risks for uteroplacental insufficiency, intermittent decreases in oxygen delivery to the intervillous space that occur during uterine contractions are usually well compensated for during the labor course. If a previously well‐oxygenated, term fetus develops clinically significant acidemia during labor, recurrent FHR decelerations will be present, and the baseline FHR variability will change from moderate to minimal and, eventually, absent. This pattern of recurrent decelerations that become progressively deeper with progressively diminished variability is sometimes accompanied by fetal tachycardia.^38^ In general, the progression from adequate oxygenation to acidemia takes approximately 1 hour.^25^ Alternatively, acute hypoxic events that do not resolve will be evident as acute fetal bradycardia. Thus, FHR characteristics detectable via EFM or IA must be interpreted with regard to any accompanying risk for the presence of fetal acidemia.

## INTERPRETATION OF THE FETAL HEART RATE VIA INTERMITTENT AUSCULTATION

A workgroup of the National Institute of Child Health and Human Development (NICHD) and American College of Obstetricians and Gynecologists recommended a 3‐tier system of categories for interpretation of FHR patterns based on the relationship between the FHR pattern captured electronically and the risk for associated fetal acidemia.^25^ This workgroup convened in 2008, and the originally proposed categories using I, II, and III remain the preferred system for electronic FHR interpretation. Prior guidance has been provided suggesting that auscultated FHR patterns can be interpreted as either Category I or Category II, consistent with the NICHD 3‐tier system of categories.^25^, ^39^ To date, there is no published research documenting validity and reliability between the use of the NICHD categories of FHR patterns (eg, Category I, II, III) and their application to FHR patterns heard with IA. Prior guidance suggesting a relationship between the patterns has not been substantiated by any research to date; therefore, it is no longer recommended to follow this guidance. Instead, FHR characteristics obtainable via IA should be documented (eg, baseline rate; accelerations/increases; decelerations/decreases, but not type of deceleration; irregular rhythm) to facilitate clinical management and documentation^7^ (see Table 4).

**Table 4:** Intermittent Auscultation Fetal Heart Rate Findings

**FHR characteristics associated with adequate oxygenation**
Normal FHR baseline between 110 and 160 bpm, regular rhythm, and absence of FHR decreases or decelerations from baseline
FHR increases or accelerations from baseline may or may not be present.
If present, FHR accelerations signify fetal well‐being at the time they are noted.
**FHR characteristics requiring additional assessment**
Irregular rhythm
Presence of FHR decreases or decelerations from baseline
Tachycardia (baseline >160 bpm for ≥10 min in duration)
Bradycardia (baseline <110 bpm for ≥10 min in duration)

Abbreviations: bpm, beats per minute; FHR, fetal heart rate.

## DOCUMENTATION

While performing IA, the following characteristics should be documented: uterine activity pattern, counted FHR, rhythm, and the presence or absence of accelerations/increases or decelerations/decreases. Terms used for each characteristic should be consistent with the terminology defined by the NICHD, where applicable to auscultated findings (Table 5).^25^, ^39^ If decelerations are noted, documentation should include the nadir rate, whether the decelerations are recurrent or nonrecurrent, and any interventions instituted (eg, position changes, hydration, clinical assessment of labor progress, increased frequency of IA, or transition to continuous EFM).^40^ If accelerations are documented, they can be detected using the criteria for identifying accelerations via auscultation that were validated by Paine et al.^17^, ^18^ In addition, information about the labor course or status of the laboring person that may assist in the interpretation of data by independent observers should be documented in the record, including FHR response to rupturing of membranes, position changes during labor, response to scalp stimulation, or results of vaginal examinations.

**Table 5:** National Institute of Child Health and Human Development Terminology for Fetal Heart Rate Characteristics Determined by Electronic Fetal Heart Rate Monitoring

Term	Definition
**Baseline rate**	Mean FHR rounded to increments of 5 bpm during a 10‐min segment excluding periodic or episodic changes, periods of marked variability, and segments of baseline that differ by >25 bpm. Duration must be ≥2 min.
**Bradycardia**	Baseline rate of <110 bpm for ≥10 min
**Tachycardia**	Baseline rate of >160 bpm for ≥10 min
**Variability**	Fluctuations in the baseline FHR of ≥2 cycles/min; this is visually quantitated as the amplitude of the peak‐to‐trough fluctuation in bpm.
Absent	Amplitude from peak to trough is undetectable.
Minimal	Amplitude from peak to trough is greater than undetectable and ≤5 bpm.
Moderate	Amplitude from peak to trough is 6–25 bpm.
Marked	Amplitude from peak to trough is >25 bpm.
**Acceleration**	Visually apparent, abrupt (onset to peak is <30 s) increase in FHR above baseline. Peak is ≥15 bpm above the baseline rate. Duration is ≥15 s and < 2 min. In gestations <32 wk, an acceleration is defined as having a peak of ≥10 bpm above the baseline and a duration of ≥10 s.
Prolonged	Acceleration >2 min and <10 min in duration
**Decelerations**	
Early	Visually apparent, gradual (onset to nadir is ≥30 s) decrease in FHR below baseline. Return to baseline associated with a uterine contraction. The nadir of deceleration occurs at the same time as the peak of the contraction. Generally, the onset, nadir, and recovery of the deceleration occur at the same time as the onset, peak, and recovery of the contraction.
Late	Visually apparent, gradual (onset to nadir is ≥30 s) decrease in FHR below baseline. Return to baseline is associated with a uterine contraction. The nadir of deceleration occurs after the peak of the contraction. Generally, the onset, nadir, and recovery of the deceleration occur after the onset, peak, and recovery of the contraction.
Variable	Visually apparent, abrupt (onset to nadir is <30 s) decrease in FHR below baseline. Decrease is ≥15 bpm. Duration is ≥15 s and <2 min.
Prolonged	Visually apparent decrease in FHR below baseline. Decrease is ≥15 bpm. Duration is ≥2 min but <10 min.

Abbreviations: bpm, beats per minute; FHR, fetal heart rate.

Adapted from Macones et al.^25^

## PATIENT SATISFACTION AND CHOICE

In a systematic review of factors contributing to laboring persons’ satisfaction with the experience of childbirth (N = 45,000), investigators identified the 4 most important factors: personal expectations, amount of caregiver support, quality of caregiver support, and involvement in decision‐making.^41^, ^42^ Involving low‐risk laboring people in shared decision‐making and offering informed choice about mode of fetal monitoring may further add to their satisfaction with the experience of labor and birth.^42^, ^43^ Since IA requires a ratio of 1 health care provider to 1 laboring person and near‐constant presence in order to perform auscultation at the recommended frequency, this method of fetal surveillance during labor may also contribute to improved outcomes and increased patient satisfaction.^44^ Last, IA allows the laboring and birthing person to move freely, which may increase positive perceptions of the birth experience.^42^, ^45^, ^46^, ^47^

## CHALLENGES AND FUTURE RESEARCH

More research is necessary to determine the most effective frequency of auscultation, inter‐ and intraobserver reliability, and barriers to the use of IA in labor. Additionally, validation of the use of the NICHD categories for interpretation of FHR patterns obtained by IA is absent from the literature. Further research into defining “low risk” for the purposes of fetal assessment would lead to clearer guidelines for appropriate candidates for IA. Given that EFM use in healthy, low‐risk laboring people is standard, in conjunction with the high rate of cesarean births in the United States, further investigations to identify clinical and nonclinical barriers to the evidence‐based use of IA are warranted.^48^ Investigatiors should also evaluate practical methods of incorporating IA into all perinatal health care provider and nursing education programs and intrapartum units. Health care providers and students need opportunities to learn and become comfortable with using IA.^35^, ^49^, ^50^, ^51^

## RECOMMENDATIONS FOR PRACTICE

In Canada and the United Kingdom, IA is the preferred method of fetal surveillance in people who enter labor at term without medical or obstetric conditions associated with uteroplacental insufficiency and/or conditions associated with an increased risk for fetal acidemia.^9^, ^10^ The American College of Obstetricians and Gynecologists indicates that for monitoring people in labor, IA is “acceptable in a patient without complications.”^8^ The frequency of observations required to monitor fetal status with IA facilitates other evidence‐based labor support practices (eg, continuous support) and should be the preferred approach to FHR monitoring. Compared with EFM, IA is associated with fewer cesarean and operative vaginal births, procedures that have additional attendant risks for the birthing person and newborn. In addition, equivalent neonatal outcomes are associated with the use of IA and EFM.^4^, ^21^, ^22^, ^23^ Finally, IA allows laboring people more mobility, which in turn increases comfort and promotes progress of labor.^42^

### Summary of Recommendations

1. IA is the preferred method for monitoring the FHR during labor at term where the risk of fetal acidemia is rare.
2. IA should be conducted according to practice guidelines that include criteria for the use of IA, criteria for converting to EFM, and protocols for frequency of observation and documentation.
3. Multiple‐count methods (eg, listen for 6 seconds, multiply by 10, note FHR, and then repeat multiple times) appear to be more accurate and reliable than are single‐count methods for evaluation of periodic changes.
4. Listening through a contraction instead of listening between contractions will likely improve the detection of periodic or episodic changes that might suggest when conversion to EFM is appropriate.
5. Auscultated FHR characteristics should be documented using approved terminology and along with descriptive notations of the labor, as needed.
6. Further research is needed to determine the reliability and validity of different IA protocols and barriers to the use of IA as a standard of care for people who enter labor with low risk of fetal acidemia.

## CONFLICT OF INTEREST

The authors have no conflicts of interest to disclose.

Copyright © 2026 by the American College of Nurse‐Midwives. All rights reserved. This bulletin may not be re‐produced or transmitted in any form by any means, electronic or mechanical, including photocopying, recording, or by an information storage and retrieval system without permission in writing from the American College of Nurse‐Midwives.

American College of Nurse‐Midwives

PO Box 905

Richmond Hill, GA, 31324

www.midwife.org

Approved: ACNM Board of Directors, October 2025.

Suggested citation: Ostrem‐Niemcewicz K, Holm J, Kane Low, L. American College of Nurse‐Midwives Clinical Bulletin. Intermittent auscultation for intrapartum fetal heart rate surveillance. *J Midwifery Womens Health*. 2026;71(x):1‐8. doi:10.1111/jmwh.70079

## Data Availability

Data sharing not applicable to this article as no datasets were generated or analysed during the current study.
